# Development and validation of a follow-up methodology for a randomised controlled trial, utilising routine clinical data as an alternative to traditional designs: a pilot study to assess the feasibility of use for the BladderPath trial

**DOI:** 10.1186/s40814-020-00713-y

**Published:** 2020-10-31

**Authors:** Harriet P. Mintz, Amandeep Dosanjh, Helen M. Parsons, Ana Hughes, Alicia Jakeman, Ann M. Pope, Richard T. Bryan, the BladderPath trial management group, Nicholas D. James, Prashant Patel

**Affiliations:** 1 Warwick Medical School, University of Warwick, Coventry, CV4 7AL UK; 2 University Hospitals Birmingham NHS Foundation Trust, Edgbaston, Birmingham, B15 2GW UK; 3 Institute of Cancer and Genomic Sciences, University of Birmingham, Edgbaston, Birmingham, B15 2TT UK; 4 The Institute of Cancer Research, 237 Fulham Road, London, SW3 6JB UK; 5 The Royal Marsden NHS foundation Trust, Fulham Road, Chelsea, London, SW3 6JJ UK

**Keywords:** Bladder cancer, Routine, Administrative, Data, Outcomes, Events, Follow-up, Randomised controlled trial (RCT), BladderPath, Hospital episode statistics (HES)

## Abstract

**Background:**

Bladder cancer outcomes have not changed significantly in 30 years; the BladderPath trial (Image Directed Redesign of Bladder Cancer Treatment Pathway, ISRCTN35296862) proposes to evaluate a modified pathway for diagnosis and treatment ensuring appropriate pathways are undertaken earlier to improve outcomes. We are piloting a novel data collection technique based on routine National Health Service (NHS) data, with no traditional patient-Health Care Professional contact after recruitment, where trial data are traditionally collected on case report forms. Data will be collected from routine administrative sources and validated via data queries to sites. We report here the feasibility and pre-trial methodological development and validation of the schema proposed for BladderPath.

**Methods:**

Locally treated patient cohorts were utilised for routine data validation (hospital interactions data (HID) and administrative radiotherapy department data (RTD)). Single site events of interest were algorithmically extracted from the 2008–2018 HID and validated against reference datasets to determine detection sensitivity. Survival analysis was performed using RTD and HID data. Hazard ratios and survival statistics were calculated estimating treatment effects and further validating and assessing the scope of routine data.

**Results:**

Overall, 829/1042 (sensitivity 0.80) events of interest were identified in the HID, with varying levels of sensitivity; identifying, 202/206 (sensitivity 0.98; PPV 0.96) surgical events but only 391/568 (sensitivity 0.69; PPV 0.95) radiotherapy regimens. An overall temporal quality improvement trend was present: detecting 41/117 events (35%) in 2011 to 104/109 (95%) in 2017 (all event types). Using the RTD, 5-year survival rates were 43% (95% CI 25–59%) in the chemoradiotherapy group and 30% (95% CI 23–36%) in the radiotherapy group; using the HID, the 5-year radical cystectomy survival rate was 57% (95% CI 50–63%).

**Conclusions:**

Routine data are a feasible method for trial data collection. As long as events of interest are pre-validated, very high sensitivities for trial conduct can be achieved and further improved with targeted data queries. Outcomes can also be produced comparable to clinical trial and national dataset results. Given the real-time, obligatory nature of the HID, which forms the Hospital Episode Statistics (HES) data, alongside other datasets, we believe routine data extraction and validation is a robust way of rapidly collecting datasets for trials.

**Supplementary Information:**

**Supplementary information** accompanies this paper at 10.1186/s40814-020-00713-y.

## Background

Outcomes for Bladder cancer have not changed significantly for decades. We hypothesise one reason for this is the delay from diagnosis to the correct treatment. The BladderPath trial (ISRCTN35296862) is assessing a redesigned pathway, replacing transurethral resection of bladder tumour (TURBT) with initial magnetic resonance imaging (MRI), with the purpose of fast-tracking patients with muscle invasive bladder cancer (MIBC) directly to the correct treatment [1].

In order to achieve broad recruitment with minimal clinical disruption, the trial also aims to use routine administrative data as the basis for follow-up. We believe that no interventional randomised controlled trial (RCT) has been conducted in an oncology setting in the UK, using routine data sources as a replacement for conventionally collected follow-up data.

**Fig. 1:**
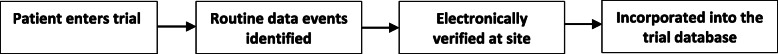
Proposed data flow for the BladderPath trial

Traditionally, upon entering a trial, data are collected during patient follow-up visits, via patient-clinician contact and case report forms (CRF) are completed manually. However, our proposed method of follow-up proceeds as shown in Fig. 1. Upon entering the trial, participant consent to access routine National Health Service (NHS) datasets (for example, Hospital Episode Statistics (HES) [2], the national radiotherapy data set (RTDS) [3] and the systemic anti-cancer therapy data set (SACT) [4]) is being obtained. These data records will be processed regularly to identify events of interest. These events will be collated into pre-populated electronic CRFs and sent to sites for verification of accuracy and completeness. The completed record will then be uploaded into the trial database.

Here, we outline a study assessing the feasibility of this proposed methodology, utilising routine data, for clinical trial follow-up within the BladderPath trial.

Specific feasibility objectives to be addressed include (1) assessing the scope of using routine data solely for RCT follow-up—for example, assessing, data quality, the availability of key variables, datasets required, routine data utility, data timeliness, regulatory requirements and designing an algorithm for data extraction. Routine data quality and utility are analysed directly by comparison to reference data sets and indirectly, through performing survival analyses; (2) If this data is deemed appropriate, design a framework for use in an RCT.

## Methods

### Data sources

The BladderPath trial proposes to use HES [2] and RTDS [3] for data collection, therefore, the local Hospital Interaction Data (HID) (returned centrally to form the HES) and RTD (local administrative linear accelerator (LINAC) machine prescription radiotherapy data with similarity to the RTDS) were used as equivalents.

Hence, five unique data sources were accessed within the University Hospitals Birmingham Queen Elizabeth Hospital (UHB QEH: BladderPath lead site); (1) RTD (reference cohort identified using the International Classification of Diseases (ICD) ICD-10 [5] bladder cancer code C67X), (2) manually collated surgical data (used for surgical cohort identification), (3) HID (inpatient and outpatient service interactions) [6], extracted using NHS number and local hospital unit number identified in the reference cohorts. The cystectomy cohort HID were extracted for events one-year prior to cystectomy date in the manually collected surgical reference and censored at 31 March 2018. The radiotherapy cohort HID were extracted from first radiotherapy event in the RTD reference, 01 January 2011 and censored at 31 May 2018, (4) clinical note review data and (5) NHS Spine data [7] for date of death. In addition, the national dataset from the British Association of Urological Surgeons (BAUS) [8], was accessed to enhance the surgical reference data where required [9]. During the validation process, these five data sources were utilised as two data types, reference (to validate) and test (to be validated) (Table 1).

**Table 1:** The reference and test datasets analysed

Data type	Dataset	Purpose	Extracted from	How cohort extracted
**Reference data**	Surgical data	-Identify cohort -Validate HID	Data quality analyses: UHB QEH (single site) Survival analyses: UHB (two sites)	Manually
	Clinical note review data	-Validate HID		Manually
	RTD (radiotherapy data)	-Identify cohort -Validate HID		By ICD-10 code
**Test data**	RTD (radiotherapy data)	-To be validated (during survival analyses^a^)		By ICD-10 code
	HID (inpatient and outpatient interactions)	-To be validated (during data quality and survival analyses^a^)		By NHS and hospital numbers (from reference)

^a^ NHS spine data was used in addition to enable survival analyses calculation

### Reference data

Reference data consisted of three sources: (1) manually collated surgical data (to validate surgical HID events), (2) RTD (to validate radiotherapy HID events) and (3) clinical note review data (to validate the following HID events: chemotherapy, cystoscopy, Bacillus Calmette-Guérin (BCG) and censor (last follow-up) (Table 1). The data analysts had extensive experience of the reference extraction processes and datasets and clinical guidance was sought (via NJ, PP and AD) where required. These were deemed suitable reference datasets due to the method of generation; the surgical and clinical note review data were collated manually by healthcare professionals and the RTD data are collected directly from radiotherapy treatment machines, upon radiotherapy administration.

### Test data

Test data consisted of two sources: HID and RTD. The RTD was used as a reference for HID quality validation but during survival analysis was also used as a test dataset alongside the HID and NHS Spine data records. Therefore, the RTD were used as both a reference and a test dataset (Table 1).

### Patient cohorts

Data quality was established using various cohorts, including 206 patients undergoing cystectomy (bladder removal) surgery (not exclusively for bladder cancer) between 08 January 2010 and 07 April 2017. Random HID identified subsets were further used to evaluate occurrences of events of interest: chemotherapy (40 patients, 47 regimen events), cystoscopy pre and post cystectomy (29 patients, 106 events), BCG (30 patients, 114 events, 15 regimen events) and last follow-up censor event (related patient visit to hospital, see Additional file 1: 100 patients, 100 events). During survival validation, 335 patients undergoing radical cystectomy were evaluated, treated between 01 January 2011 and 07 April 2017 (132/335 surgical events involved in data quality assessment above and 203/335 novel events, including patients from other sites within UHB. The remaining 74/206 patients used in the data quality assessment were excluded due to: 4 not coded for cystectomy, 18 partial cystectomies, 14 prior to 01 January 2011 and 38 performed for non-bladder cancer purposes). The patients were identified from the HID data using OPCS-4 [10] cystectomy codes (Additional file 1). ICD-10 (C67 bladder cancer and D090 bladder cancer in situ) codes were used to identify bladder cancer where case note review reference was not possible (e.g. for non-UHB QEH).

In addition, 525 bladder cancer patients were identified from the RTD (radical and palliative), treated between 01 January 2011 and 11 June 2018, of which 524 had at least one HID event. 336/525 of the patients, who were undergoing radical radiotherapy (identified by the LINAC defined intention to treat) were further evaluated with respect to survival outcomes. For data quality validation, a total of 707 patients had at least one event of interest validated.

### Processing and outcome measures

An algorithm was written in R [11] using R Studio [12] to extract events of interest from the routine HID. Events of interest: surgery to bladder to remove tumour (cystectomy, cystoprostatectomy, exenteration), radiotherapy (radical, palliative), cystoscopy (all cystoscopies, including but not limited to flexible (cystoscopy or urethroscopy) or rigid TURBT), BCG therapy, chemotherapy (any cancer) and last known interaction with urology or oncology services (inpatient or outpatient event censor).All procedures were identified using Classification of Interventions and Procedures (OPCS) version 4.4–4.8 (10) and censor date validated from the NHS Digital main speciality coding [13] (Additional file 1). For survival analysis, algorithms were written using the Microsoft SQL server; for radical radiotherapy outcomes, synchronous chemotherapy events were extracted from the HID (Additional file 1, code present 4 weeks ± radiotherapy initiation) and linked to the RTD and NHS Spine data sets. For radical cystectomy outcomes, the cystectomy-type procedures were identified in the HID using OPCS codes (Additional file 1). For maximum data follow-up (90 months), survival outcome analyses (post-01 January 2011), cystectomy participants without a survival event were censored at the data freeze, 14 June 2018, and radiotherapy participants, 29 June 2018.

### Sample size summary

Two cohorts were extracted for the analyses (radiotherapy and surgical). Initially the cohorts were searched for all patients receiving radical or palliative radiotherapy for bladder cancer (525 patients), or surgery to the bladder (277 patients), treated at UHB between January 2011–June 2018 and January 2010–April 2017, respectively. Subsets were analysed for the data quality and survival analyses, which can be seen in detail in the ‘patient cohorts’ subsection above.

### Analytical methods

The events of interest, by date, were manually compared to the reference events, and sensitivity and positive predictive value (PPV) calculated. Concordance of exact procedure code was not required due to the querying technique not requiring exact identification for the BladderPath trial. Operation dates were not available for outpatient events (e.g. flexible cystoscopy); therefore, date of appointment was validated. For radiotherapy, chemotherapy and BCG events, analysis was undertaken for regimen level accuracy, detection of only one event was required to identify the regimen. Events were subsequently grouped by year to assess sensitivity over time. Kaplan-Meier survival curves were constructed using Stata version 15 [14] to identify 5 -year (60 months) survival plus 95% confidence intervals (CI), comparing chemoradiotherapy and radiotherapy alone or radical cystectomy. Cox proportional hazards models were constructed for radiotherapy hazard ratio (HR) analyses with 60, 72 and 90-month follow-up. Patient characteristics were calculated using the routine HID, including the Charlson Comorbidity Index scores (Charlson scores) [15] identified upon inpatient procedure (either the date of surgery to bladder or the nearest inpatient admission to the start date of the radiotherapy).

## Results

Patient characteristics can be seen below in Table 2. Only one patient (radiotherapy data quality cohort, palliative) had no coded HID events; hence, the number of patients included in the analysis were 524/525 (99.8%).

**Table 2:** Patient characteristics for the surgical and radiotherapy cohorts

		Data quality analysis cohorts		Survival analysis cohorts		
		All radiotherapy	Surgical	Radical radiotherapy		Radical cystectomy
		***n*** = 525	***n*** = 206	Chemoradiation ***n*** = 66	Radiotherapy alone ***n*** = 270	All ***n*** = 335
		Number of patients (%)				
**Age at 1st treatment**	**Median (IQR)**	75 (68–94)	66.5 (56–73)	75 (67–79)	76.5 (70–82)	68 (62–74)
	**Range**	31–96	22–85	52–90	42–94	23–86
**Gender**	**Male**	380 (72.4%)	147 (71.4%)	47 (71.2%)	200 (74.1%)	248 (74.0%)
	**Female**	144 (27.4%)	59 (28.6%)	19 (28.8%)	70 (25.9%)	87 (26.0%)
**Ethnicity**	**White**	401 (76.4%)	191 (92.7%)	53 (80.3%)	203 (75.2%)	304 (90.7%)
	**Asian/Asian British**	18 (3.4%)	9 (4.4%)	2 (3.0%)	9 (3.3%)	17 (5.1%)
	**Black/Black British**	5 (1.0%)	3 (1.5%)	0 (0.0%)	4 (1.5%)	1 (0.3%)
	**Mixed**	7 (1.3%)	0 (0.0%)	2 (3.0%)	3 (1.1%)	1 (0.3%)
	**Other**	2 (0.4%)	2 (1.0%)	1 (1.5%)	0 (0.0%)	2 (0.6%)
	**Unknown**	91 (17.3%)	1 (0.5%)	8 (12.1%)	51 (18.9%)	10 (3%)
**Charlson score**	**< 1**	253 (48.2%)	116 (56.3%)	33 (50.0%)	125 (46.3%)	216 (64.5%)
	**1–5**	73 (13.9%)	45 (21.8%)	14 (21.2%)	31 (11.5%)	55 (16.4%)
	**6–10**	39 (7.4%)	23 (11.2%)	8 (12.1%)	17 (6.3%)	45 (13.4%)
	**11–15**	21 (4.0%)	10 (4.9%)	3 (4.5%)	7 (2.6%)	12 (3.6%)
	**16–20**	11 (2.1%)	5 (2.4%)	4 (6.1%)	5 (1.9%)	4 (1.2%)
	**> 20**	8 (1.5%)	3 (1.5%)	0 (0.0%)	4 (1.5%)	3 (0.9%)
	**Unknown**	119 (22.7%)	4 (1.9%)	4 (6.1%)	81 (30.0%)	0 (0.0%)

Patients without a HID inpatient event have an unknown Charlson score. Due to rounding, percentages may not sum to 100%

### Data quality

Overall, 829/1042 (sensitivity 0.80) events were identified in the HID (Additional file 2), with the individual events by year seen in Table 3. There was an overall data quality improvement of 60.4% (2011–2017), from detecting 41/117 (2011 sensitivity 0.35) to 104/109 events (2017 sensitivity: 0.95), with a mean sensitivity of 0.97 over the last 4 years (Additional file 3).

**Table 3:** Sensitivity of the HID coding compared to the reference events, over a 10-year period (2008–2018)

	Number of events	Year of event											No. false positives (PPV)
		2008	2009	2010	2011	2012	2013	2014	2015	2016	2017	2018	
Cystectomy	Reference	NA	NA	16	21	28	26	41	34	34	6	NA	8 (0.96)
	Routine data (HID)	NA	NA	16	21	25	25	41	34	34	6	NA	
	Sensitivity (%)	**–**	–	100.0	100.0	89.3	96.2	100.0	100.0	100.0	100.0	-	
Radiotherapy regimen	Reference	NA	NA	NA	74	83	72	67	79	93	68	32	20 (0.95)
	Routine data (HID)	NA	NA	NA	1	1	55	66	79	92	68	29	
	Sensitivity (%)	**–**	–	–	1.4	1.2	76.4	98.5	100.0	98.9	100.0	90.6	
Censor	Reference	NA	NA	1	5	4	8	16	11	9	27	19	0 (1.00)
	Routine data (HID)	NA	NA	1	5	4	8	16	11	9	22	13	
	Sensitivity (%)	**–**	–	NA	100.0	100.0	100.0	100.0	100.0	100.0	81.5	68.4	
BCG regimen	Reference	NA	NA	1	3	1	4	2	3	1	NA	NA	20 (0.41)
	Routine data (HID)	NA	NA	1	3	0	4	2	3	1	NA	NA	
	Sensitivity (%)	**–**	–	NA	100.0	–	100.0	100.0	100.0	–	–	–	
Cystoscopy	Reference	NA	3	8	8	21	20	15	15	9	5	2	6 (0.94)
	Routine data (HID)	NA	2	6	6	19	17	12	11	9	5	2	
	Sensitivity (%)	**–**	66.7	75.0	75.0	90.5	85.0	80.0	73.3	100.0	100.0	100.0	
Chemotherapy regimen	Reference	1	1	4	6	7	9	5	7	3	3	1	3 (0.94)
	Routine data (HID)	0	0	4	5	7	9	5	7	3	3	1	
	Sensitivity (%)	**–**	–	100.0	83.3	100.0	100.0	100.0	100.0	100.0	100.0	–	

NA = no events were validated (due to the random sample selected, or due to the data censor, for example, surgery to bladder censor mid-2017). (-), if only one event, or none were validated, the sensitivity was not calculated due to the sample size. Only six cystectomies were validated in the 2017 data due to the reference data freeze

In the surgical cohort, 206/206 patients had at least one inpatient or outpatient interaction identified in the HID (sensitivity 1.00). 202/206 (sensitivity 0.98) surgical events were identified less than 2 weeks from the reference date of procedure (delays included, two 1-day, one 4-day and one 13-day delay). Therefore, 198/206 (sensitivity 0.96) procedures were identified to the exact date. Eight false positives were detected (PPV 0.96) due to duplicates, unrelated and abandoned procedures. The coding quality was consistently high with the greatest number of missing events in 2012 (three). 44/47 (sensitivity 0.94) chemotherapy regimens were identified with three false positives (BCG treatments) (PPV 0.94); again the detection rate was consistently high (2010–2017) with all events captured post-2011. 89/106 cystoscopies (sensitivity: 0.84), including 32/32 (sensitivity 1.00) TURBT and 41/53 (sensitivity: 0.77) flexible cystoscopy events, were identified, plus six false positives (PPV 0.94) (nephrostogram plus insertion of stent, cystodiathermy, three duplicate records and an extirpation of bladder lesion). 89/100 (sensitivity 0.89) censor events were identified, with a decrease in data quality post-2016 (in contrast to other outcomes). 114/149 (sensitivity 0.77) individual BCG administrations and 14/15 regimens were identified (sensitivity: 0.93), with 20 false positive regimens (PPV 0.41) (the majority due to Mitomycin C administration).

In the radiotherapy cohort, 524/525 patients had at least one inpatient or outpatient interaction in the HID (sensitivity 1.00). 391/568 (sensitivity 0.69) of regimens were identified, with 20 false positives (PPV 0.95). Data quality improved by 98.6% between 2011 (sensitivity 0.01) and 2017 (sensitivity 1.00). 5121/7894 individual fractions (sensitivity 0.65) were identified.

**Fig. 2:**
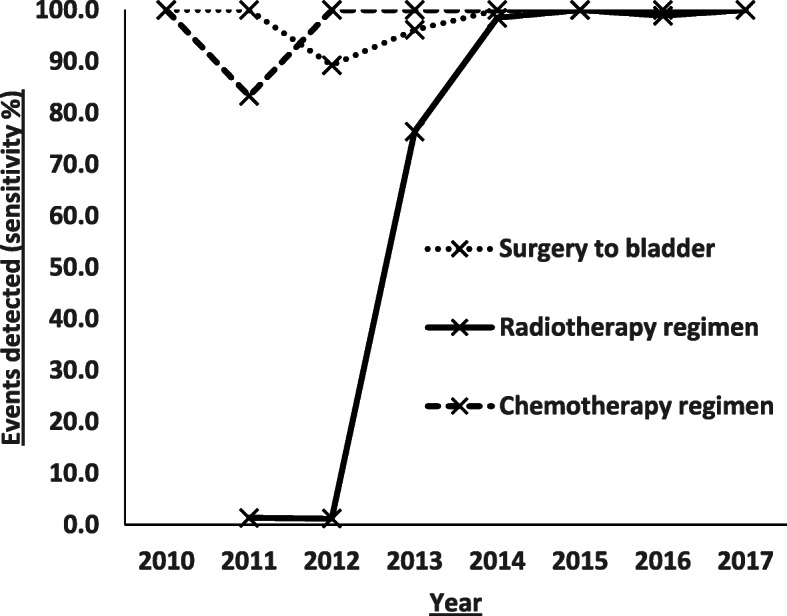
Sensitivity of detection for the first correct treatments collected as primary outcome measures in BladderPath (surgery to bladder, chemotherapy and radiotherapy) for HID data years 2010–2017

The sensitivity of detecting the main correct treatments (surgery, radiotherapy and chemotherapy), enabling calculation of BladderPath primary outcome measures, can be seen in Fig. 2.

### Survival analysis

**Fig. 3:**
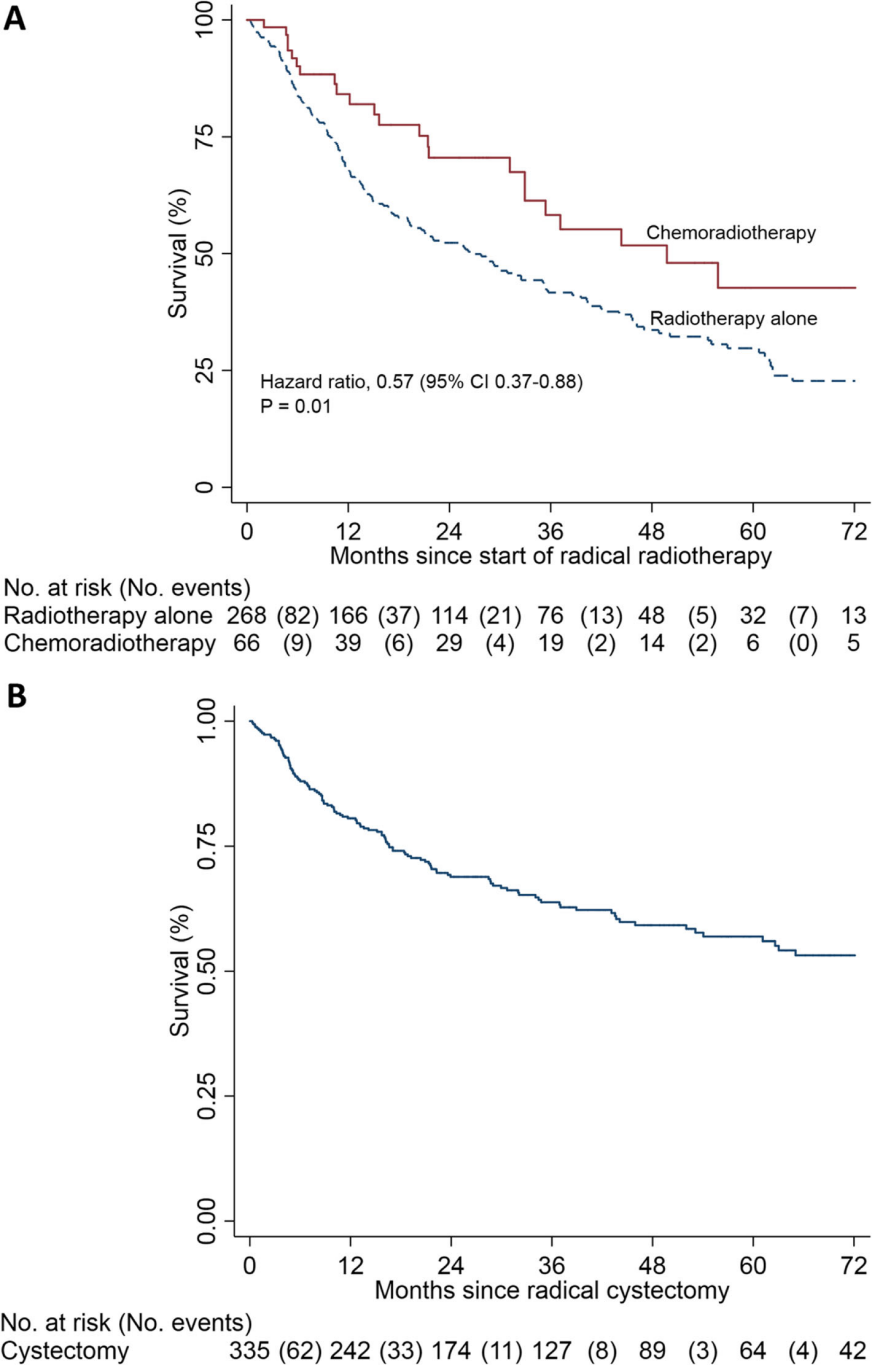
Kaplan Meier survival curves for the routine data (HID & RTD) derived data cohorts to 6 years. **a** Radiotherapy outcomes, showing 6-year HR. **b** Cystectomy outcomes

In the RTD analysis, 5-year survival rates (Fig. 3) were 43% (95% CI 25-59%) in the chemoradiotherapy group compared to 30% (95% CI 23–36%) in the radiotherapy group alone (hazard ratio, 0.57 with 6-year (72 months) follow-up (95% CI 0.37–0.88; *P* = 0.01)). In the HID cystectomy analysis, the 5-year cystectomy survival rate was 57% (95% CI 50–63%). By comparison to published trial and national datasets, the routine data integrity and utility is indirectly validated and hence, further provides evidence towards the feasibility of using the RTDS and HES for BladderPath.

## Discussion

Clinical trials have used routine data to supplement or verify data collection for decades [16–18] and many data validation studies have been undertaken into different databases worldwide [19, 20]. The benefits and limitations of utilising routine data for RCTs have also been evaluated in depth [21–24] but, despite this, there is limited evidence and therefore, confidence, of using routine data as a replacement to traditional patient-Health Care Professional follow-up techniques within clinical trials [25, 26]. Most RCTs involve this clinical contact to record outcomes, which is resource, time and cost intensive; we believe the use of routine data may provide an alternative framework.

The results of this study have directly informed the data collection techniques for the BladderPath trial. As hypothesised, events are missed, but this is estimated to have little impact on the data quality for the trial. As shown, data quality is improving, with a mean sensitivity of 97% over four later years (2014–2017). Surgical coding was of consistently high quality, contrary to the radiotherapy coding which was low quality until 2013/2014. This dramatic improvement in radiotherapy coding quality occurred following coding consultation, due to the primary payment function of these administrative data, impacting remuneration for the hospital. Due to remuneration driving central and local initiatives, we postulate that this increase in accuracy would occur at other centres nationally [27]. The quality of all data items, except censor date, reached 100% in the last full HID data year (2017). The national data quality will be assessed upon acquisition of these data for BladderPath; each event of interest will be queried in the clinical noting at each site. BladderPath aims to develop a feedback mechanism to continually send this quality measure to the data providers for service improvement, aiming to remove the query requirement for future trials. As BladderPath is designed without clinic-based follow-up, the events cannot be validated against standard trial data, only clinical noting.

Of note, the three missing radiotherapy events in 2018 highlights a limitation of HID/HES data - time lag in data access, resulting in non-identifiable events occurring after the HID data censor. Until clinical systems can produce and synchronise real-time data with routine data providers, alongside continual automatic data cleaning processes, a delay will be present when acquiring routine data. This is particularly important for trials collecting safety related events. Hence, it is possible that some trials may not be appropriate to follow-up in this manner. We believe, overall, this delay may be comparable, if not improved, to conventionally obtained trial data (collected during predefined follow-up visits) particularly when visits become less frequent, upon long term follow-up. Data providers release data with different delays. Discussion is currently underway with providers to ensure that this delay is minimal; the providers understand that this technique is novel and are developing this process alongside BladderPath. The feasibility of the approach will be confirmed upon acquisition of the data.

To ensure maximum data quality, further reduce missingness and increase our confidence, we also intend to cross-check HES events with the following additional datasets: the National Radiotherapy dataset (RTDS) [3], Systemic Anti-Cancer Therapy data set (SACT) [4] and the Diagnostic Imaging Database (Table 4) [28]. Nationally (within England), these data are collected to a structured schema, so events are available in the same format across sites. Due to not having access to the national databases within this study, a restricted number of data sources were validated here. However, additional databases should increase event detection within the trial. However, the more datasets acquired, the more resources are needed to (1) apply for these data, (2) receive these data at frequent intervals, possibly from multiple providers (arranging transfer and for which participants), (3) merge these data (potentially from multiple providers with potential data updates), (4) validate these data and (5) process these data (produce meaningful CRF data). These steps require extensive planning, for example, for receiving these data; during the trial there will be cohort alterations (patients recruiting or withdrawing). Hence, for every extract BladderPath plan to send the providers an updated cohort list to re-run the data query.

**Table 4:** Direct implications of this study to BladderPath

Outcome	Database	Implication from validation
**Surgery to bladder**	- Hospital Episode Statistics (HES)	- Historically high quality - HES data alone sufficient
**Chemotherapy regimens**	- Hospital Episode Statistics (HES) - Systemic Anti-Cancer Therapy (SACT)	- Historically high quality to detect regimens - The exact date of administrations can additionally be found in the SACT data (and clinical noting if required) - HES data alone sufficient
**Radiotherapy regimens**	- Hospital Episode Statistics (HES) - National Radiotherapy Data set (RTDS)	- More recent high quality (since 2014) to detect regimens - Due to the validation of the radiotherapy LINAC data, the RTDS will be used to supplement missing events
**Cystoscopy**	- Hospital Episode Statistics (HES) - Diagnostic Imaging Data set (DID)	- Recent high quality (since 2016) - Consistent high quality TURBT coding - Historically lower quality of flexible cystoscopy coding - Prior to trial data confidence, a database query process may be necessary (check flag = if no flexible cystoscopy is identified prior to TURBT) - To confirm identification of subsequent surveillance flexible cystoscopy events, the DID will be used as a supplement
**BCG regimens**	- Hospital Episode Statistics (HES) - Systemic Anti-Cancer Therapy (SACT)	- More recent high quality (since 2013) - SACT data will supplement missing administration details
**Censor**	- Hospital Episode Statistics (HES)	- Data quality historically high, but reduced recently (post-2016) - Therefore, upon query at site, the most recent event in the clinical noting should be confirmed

The datasets analysed in this study were deemed suitable equivalents to the national datasets for BladderPath. Where alternatives are available (local data), initially national data should not be acquired as may not be fit for purpose. Alternatives enabled this proof-of-concept study prior to acquiring data for BladderPath. It is hypothesised that the HES data will exceed the HID quality due to additional provider level processing, prior to release.

The technique of querying all data items against a clinical reference as verified in this study, during the trial, will also add an additional confirmation of data integrity, acting as further data validation across multiple sites. Although, some level of missing data in trials is to be expected [29] and as we have shown previously, routine data have the ability to identify some missing trial events [30]. The above methods aim to enhance data quality and reduce missingness.

We further validated these data and showed that these routine data derived events could be used to perform analyses such as survival analysis. The radiotherapy and cystectomy survival statistics are comparable to published clinical trial results [31] and national datasets respectively [32], further establishing the utility of both the RTDS and HES for the trial and establishing data integrity. During the RTD analysis, HRs were also constructed at 5 years and to the end of the study period (90 months) (Additional file 4). Comparison with clinical trial results should be interpreted with caution due to the non-comparable, non-randomised case-mix in our patient cohorts; likewise, comparisons cannot be drawn between radiotherapy and cystectomy outcomes due to heterogeneity.

The algorithm is designed to capture as many events as possible, requiring an exceptionally high sensitivity. Therefore, additional codes are identified for unrelated procedures (marked in Additional file 1) that may have been incorrectly coded. For this reason, a lower PPV was acceptable, although, a lower PPV will result in greater burden on site staff validating false positive events, so a balanced approach is required. It is not possible to calculate the specificity or the negative predictive value as the number of true negatives is not known; we did not have access to a reference identifying patients that did not have events. However, as each event will be queried and confirmed before incorporation into the trial database, by definition the trial event specificity will be 100%. For treatments with regimens (radiotherapy, chemotherapy and BCG), it is only necessary to flag one instance of administration per regimen to identify these outcomes, as further targeted details can be extracted from the clinical noting. Hence, we have shown the primary outcome for the intermediate stage of BladderPath (time to correct treatment for all possible MIBC patients) can be feasibly identified in routine data.

Limitations of this feasibility study include single site analysis, except for the cystectomy survival analysis where patients from two hospital sites were analysed. Implications of these include, coding inconsistencies, if any, and missed events. As shown, the ability of the HID data to replicate results using national datasets [32], suggests that our sample may be representative of multiple sites. Lack of data from other hospitals also resulted in missing Charlson scores, as inpatient admissions occurred at different sites to the radiotherapy. Although a limitation for this paper, as discussed above, we do not envisage a similar issue in the BladderPath trial, as we will have data access across all English sites.

Another data limitation involves the lack of clinical/pathological event level data in the HID, which has implications to the interpretation of the survival analysis, limiting the statistical control of the heterogeneity in the comparisons. The strongest predictors of bladder cancer survival include, but are not limited to, pathological patterns (tumour grade, stage and lymph node involvement), histologic patterns (lymphovascular invasion), demographic and epidemiological characteristics (gender, age) and clinical characteristics (neutrophil-lymphocyte-ratio) [33]. Further predictors include preoperative (neoadjuvant) chemotherapy [34], Charlson score [35] and soft tissue surgical margins [36] (Table 5). Of these, gender, age, Charlson score and preoperative neoadjuvant chemotherapy are identifiable in administrative data and as such were analysed in this analysis. The remaining variables were not present in the HID, but the majority can be collected using cancer registries (Table 5). Prior to performing survival analysis within a trial setting, all required variables should be validated for completeness and accuracy.

**Table 5:** The strongest predictors of bladder cancer survival and whether these variables can be theoretically identified from administrative or registry data, in the absence of clinical trial data

Variable	Administrative	Registry
Gender^a^	✓	✓
Age^a^	✓	✓
Neutrophil-lymphocyte-ratio	✗	✗
Lymphovascular invasion	✗	?
Tumour stage and grade	✗	✓
Lymph node involvement	?	✓
Neoadjuvant chemotherapy^a^	✓	✓
Charlson score^a^	✓	✗
Surgical margins	✗	✓

^a^Used in survival analyses. Administrative classified as HID (HES), RTDS, SACT; registry classified as cancer registration (not including individualised cancer registries) ✓ = theoretically collected, *✗* = not collected, ? = not explicitly collected (e.g. histology coded and free text field available, but lymphovascular invasion not collected individually, secondary lymph node involvement can be coded in diagnosis fields using ICD coding, but not obligatory)

In addition to a lack of fields at event level, routine data can be limited at patient level; HES are collected for NHS patients and not for private care; thus, these events would be missed. Many datasets are also restricted by location; HES are only collected for England. However, there are alternatives but the practical burden (performing the processes mentioned above) will increase upon acquisition of multiple data sources. However, in the absence of non-English data, the BladderPath framework ensures that follow-up can continue using clinical noting. This has been tested at multiple sites within BladderPath and is feasible; this can be seen in the excellent CRF data completion rates. In addition, the ability to query ensures that unavailable data variables (for example, missing or limited by location) can be identified. Prior to other trials utilising this method, it is vital to assess if both the events and the cohort of interest are both available within these data.

Although the often arbitrary value of a reference standard has been frequently debated [37, 38], limitations were identified with regards to the reference data. Manually reviewing clinical noting can miss events occurring in other hospitals and inaccurate initial recording may also lead to inaccurate data [39], resulting in inaccurate measures of sensitivity. Additional reviewers of the reference sources were unavailable. However, the data analysts had extensive experience of these extraction processes and datasets. In addition, clinical guidance was sought (via NJ, PP and AD) where required. Hence, errors should be minimal. In addition, the radiotherapy RTD reference identified fractions prescribed, not delivered (as in the HID). Although, anecdotally at the BladderPath lead feasibility site, the prescribed and delivered relationship is extremely close; therefore, implying this would have little impact on sensitivity. This seems a reasonable assumption to extend to all other BladderPath sites, as implications of any misclassification would increase sensitivity (if regimens thought to be missed in the HID, were never delivered (only prescribed in the RTD), the number of false negatives would reduce).

Routine data-based follow-up aims to reduce costs compared to standard data collection techniques. However, if the costs are too high to receive frequent datasets from providers, these techniques become redundant. This schema is novel and therefore the data providers are keen to make this affordable.

There are also regulatory considerations. Hence, applications require continual communication with providers, ideally during trial set-up. For example, consent forms need to be designed to enable data access. Methods for optimum BladderPath data security and privacy are being discussed, including how these data will be sent, where these data will be analysed, stored and then kept (retention). Retention is essential for audit purposes and trials have to make agreements with providers.

The study aimed to identify the scope of using routine data solely for follow-up and if possible, to design a framework. The next stage is to acquire these data, validate the framework within the trial and validate events across multiple sites. We identified the following practical considerations when utilising routine data for data collection; missingness (erroneous or occurring outside of the NHS or England), accuracy, outcome availability, timeliness, costs and regulatory considerations such as privacy, security, consent and data retention. Despite these, BladderPath has confirmed the feasibility of this approach. Liaising with data providers throughout the trial set-up period is essential and helps minimise these issues.

Potential strengths of this framework include higher quality data (than if human reported), economic benefits (funds could be redistributed elsewhere), rapid updatable datasets, reduced burden on site staff (targeted data queries and semi-prepopulated CRFs) improving efficiency, traceable data changes (aiding audit trails), real-time data monitoring (dashboarding) and contact-free follow-up. These aim to be tested within BladderPath. There are well known concerns with using routine data to conduct clinical trials [24]. However, we believe this trial design mitigates these concerns by using multiple datasets to capture events and cross-correlating outcomes with targeted data queries at site.

## Conclusion

Although clinical trials have used routine data to supplement or verify data collection for many decades [16–18], to our knowledge, we believe there is limited evidence of RCTs using routine data as the primary method of patient follow-up. Furthermore, we know of no RCTs which use this technique in an oncology setting in the United Kingdom. We therefore set out, and have shown, the feasibility of this approach for use in a multi-centre study. It is possible that for the foreseeable future there will be reduced face-to-face clinical follow-up due to COVID-19. Hence, a framework such as this may facilitate oncology research during these times.

Limitations of this approach are predominantly due to data quality in the routine data repositories. However, we have shown that, over time, data quality has improved. So, whilst routine data are not yet of high enough quality to be used as a sole definitive event marker, trials can undertake an additional querying framework such as the one which we have outlined above. Hence, we believe that the BladderPath study may create a paradigm shift away from traditional trial frameworks, resulting in cheaper, less resource intensive clinical trials; despite the requirement for bespoke validated algorithms.

## Supplementary Information

**Additional file 1.** Coding. Description of data: Codes identified by the algorithm to detect events and outcomes

**Additional file 2.** Sensitivity by event. Description of data: Total events identified in the reference data, compared to the number identified in the routine data, split by event

**Additional file 3.** Sensitivity by year. Description of data: Total events in the analysis, identified in the reference data and the routine data, by year

**Additional file 4.** Hazard ratios. Description of data: Hazard ratios constructed at five and six years of follow-up, for radiotherapy outcomes. The hazard ratio using all available data (90 months), can also be seen.

## Data Availability

These data that support the findings of this study are available from University Hospitals Birmingham, but restrictions apply to the availability of these data, which were used under license for the current study. Due to ethical and legal reasons, these data cannot be made publicly available, as public availability would compromise patient confidentiality.

## Abbreviations

CRF: Case report form
NHS: National Health Service
RCT: Randomised controlled trial
HES: Hospital episode statistics
HID: Hospital interactions data
RTD: Radiotherapy data
UHB QEH: University Hospitals Birmingham Queen Elizabeth Hospital
Charlson score: Charlson Comorbidity Index score
NIHR: National Institute for Health Research
MRI: Magnetic resonance imaging
BC2001: Bladder Cancer 2001
HR: Hazard ratio
CI: Confidence interval
PPV: Positive predictive value
BCG: Bacillus Calmette-Guérin
TURBT: Transurethral resection of bladder tumour
MIBC: Muscle invasive bladder cancer
LINAC: Linear accelerator
ICD: International classification of diseases
OPCS: Classification of Interventions and Procedures
RTDS: Radiotherapy data set
SACT: Systemic anti-cancer therapy
DID: Diagnostic imaging data set
